# Draft Genome Sequence of *Blautia faecis* Strain Marseille-P328, Isolated from the Human Ascending Colon

**DOI:** 10.1128/genomeA.01383-16

**Published:** 2016-12-15

**Authors:** Davide Ricaboni, Morgane Mailhe, Noémie Labas, Véronique Vitton, Didier Raoult, Matthieu Million

**Affiliations:** a Unité de Recherche sur les Maladies Infectieuses et Tropicales Emergentes, CNRS (UMR 7278), IRD (198), INSERM (U1095), AMU, UM63), Institut Hospitalo-Universitaire Méditerranée-Infection, Faculté de Médecine, Aix-Marseille Université, Marseille, France; b Department of Biomedical and Clinical Sciences, 3rd Division of Clinical Infectious Disease, University of Milan, Luigi Sacco Hospital, Milan, Italy; c Service De Gastroenterologie, Hôpital Nord, Assistance Publique-Hôpitaux de Marseille, Marseille, France; d Special Infectious Agents Unit, King Fahd Medical Research Center, King Abdul Aziz University, Jeddah, Saudi Arabia

## Abstract

*Blautia faecis* strain Marseille P328 was isolated from the ascending colon of a French patient. We sequenced the 4.45-Mb genome of the strain and compared it with that of other species of the *Blautia* genus.

## GENOME ANNOUNCEMENT

The *Blautia* genus was created in 2008 after the description of *B. wexlerae* and the reclassification among this genus of species previously classified as *Clostridium* (*C. coccoides*) and *Ruminococcus* (*R. luti*, *R. schinkii*, *R. hansenii*, *R. productus*, and *R. hydrogenotrophicus*) (1). Species of this genus are Gram positive, coccoid or oval-shaped, and strictly anaerobic bacteria normally found in human stool samples. *Blautia* species are part of the human healthy mature anaerobic gut microbiota (2, 3). Three new *Blautia* species (*B. faecis*, *B. stercoris*, and *B. glucerasea*) were then discovered, and another *Ruminococcus* species (*R. obeum*) was moved to this genus, bringing the total number of *Blautia* species to 11 (4–7). Strain Marseille-P328 was isolated from the ascending colon of a 63-year-old French male who underwent upper and lower endoscopy during his hospitalization at La Timone Hospital, Marseilles, France, in July 2014. Prior approval has been obtained from our institutional review board for this study. Strain Marseille-P328 was identified at the species level as *Blautia faecis* on the basis of a 16S rRNA gene sequence similarity of 99.68% (1,258/1,262 bp) with *Blautia faecis* strain M25^T^ (accession no. NR_109014.1). *B. faecis* strain Marseille-P328 was deposited in the CSUR collection under number CSUR P328.

Genomic DNA was isolated from *Blautia faecis* strain Marseille-P328 and cultured on 5% sheep blood-enriched Columbia agar at 37°C in an anaerobic atmosphere. *Blautia faecis* strain Marseille-P328 genomic DNA was sequenced using the MiSeq technology with the mate-pair strategy (Illumina, Inc., San Diego, CA, USA). The 852,045 paired reads were automatically trimmed by the Illumina MiSeq software and then assembled into scaffolds using Velvet (8). The resultant genome was 4,454,129 bp long and was composed of 19 scaffolds and 28 contigs. The G+C content was 42.94%. Genome annotation was performed as previously described (9).

The sequenced genome contained 3,969 protein-coding genes and a minimum of 80 predicted RNAs, including seven 5S rRNAs, five 16S rRNAs, two 23S rRNAs, and 66 tRNAs. A total of 3,057 identified genes were assigned putative functions (by COGs or by NR blast). One hundred seventy-nine genes were identified as ORFans (4.51%). The remaining genes were annotated as hypothetical proteins (638 genes [16.07%]). The resulting coding capacity was estimated at 3,852,314 bp (86.4% of the total genome).

Then, the *B. faecis* Marseille-P328 genome was incorporated into *in silico* DNA-DNA hybridization and tested against all the *Blautia* genus NCBI available reference genomes. DDH values were estimated using the GGDC online version 2.1 (http://ggdc.dsmz.de/distcalc2.php). This analysis yielded 29.7% ± 2.45% similarity with *Blautia wexlerae* (accession no. AXVN00000000), 33.3% ± 2.45% with *Blautia obeum* (accession no. AAVO00000000), 25.3% ± 2.40% with *Blautia hansenii* (accession no. ABYU00000000), 21.9% ± 2.35% with *Blautia schinkii* (accession no. JNKJ00000000), 21.2% ± 2.35% with *Blautia hydrogenotrophica* (accession no. ACBZ00000000), 22.1% ± 2.35% with *Blautia producta* (accession no. ARET00000000), 28.00% ± 2.40% with *Ruminococcus gnavus* (accession no. JAGQ00000000), 34.4% ± 2.45% with *Ruminococcus torques* (accession no. AAVP00000000), and a 31.6% ± 2.45% with *Blautia coccoides* (accession no. BAHT00000000).

### Accession number(s).

The 16S rRNA gene and genome sequences from *B. faecis* strain Marseille-P328 (= CSUR P328) were deposited in EMBL-EBI under numbers LT223578 and FLKC00000000, respectively.
